# Characterization of Parkinson’s disease-related pathogenic TMEM230 mutants

**DOI:** 10.1080/19768354.2018.1453545

**Published:** 2018-03-22

**Authors:** Daleum Nam, Hyejung Kim, Dong-Joo Choi, Yun-Hee Bae, Byoung Dae Lee, Ilhong Son, Wongi Seol

**Affiliations:** a InAm Neuroscience Research Center, Sanbon Medical Center, College of Medicine, Wonkwang University, Gunposi, Gyeonggido, Republic of Korea; b Department of Pharmacology, School of Medicine, Ajou University, Suwonsi, Gyeonggido, Republic of Korea; c Department of Neuroscience, Graduate School, Kyung Hee University, Seoul, Republic of Korea; d Department of Physiology, School of Medicine, Kyung Hee University, Seoul, Republic of Korea; e Department of Neurology, Sanbon Medical Center, College of Medicine, Wonkwang University, Gunposi, Gyeonggido, Republic of Korea

**Keywords:** TMEM230, Parkinson’s disease, LRRK2, vesicle trafficking, neurotoxicity

## Abstract

Parkinson’s disease (PD) is the second most common neurodegenerative disease. Although most PD cases are sporadic, 5–10% of them are hereditary and several pathogenic mutations in related genes have been identified. Mutations in *TMEM230* were recently identified as a cause of autosomal dominant PD. However, the basic properties of the mutant proteins are not yet known. We examined stability and neurotoxicity, important characteristics of PD pathogenesis-related proteins, of WT TMEM230 and two pathogenic mutants, R78L and PG5ext, in a dopaminergic neuronal cell line. Our study showed that amount of protein expressed in the same vector backbone was R78L > WT > PG5ext. The stabilities of the mutant proteins were similar to each other, but lower than that of the WT. In addition, overexpression of mutants and WT TMEM230 caused similar levels of neurotoxicity upon MPP^+^ treatment when compared to the cells transfected with an empty vector. Because the proteins encoded by two PD-causing genes, *TMEM230* and *LRRK2*, function in vesicle trafficking, we tested whether they interact. LRRK2 neither interacts with, nor phosphorylates TMEM230. We also investigated the levels of several Rab proteins (Rab1A, 5, 7, 8A and 11) involved in vesicle trafficking after TMEM230 overexpression. However, there was no clear difference of any Rab proteins among cells transfected with an empty vector, TMEM230 WT and mutants-expressing cells, suggesting that TMEM230 does not directly regulate these Rab proteins. Thus, these TMEM230 PG5ext and R78L mutant proteins are not distinctly different from the WT proteins except for their stability.

**Abbreviations:** LRRK2: Leucine-rich repeat kinase 2; PD: Parkinson's disease; AD: Alzheimer's disease; RT-PCR: reverse transcription-polymerase chain reaction; SDS-PAGE: sodium dodecyl sulfate-polyacrylamide gel electrophoresis; FACS: fluorescence-activated cell sorting; PBS: phosphate buffered saline; FBS: fetal bovine serum; PI: propidium iodide.

## Introduction

Parkinson’s disease (PD) is a movement disorder, and it is the second most common neurodegenerative disease after Alzheimer’s disease (AD). Similar to AD, most cases of PD are sporadic, although 5–10% are hereditary. Through decades of study, several mutations in PD-related genes that cause PD have been identified (Martin et al. 2011). Among the numerous altered functions of PD-related gene products, impairment of protein degradation leading to protein aggregation and mitochondria dysfunction have been reported. *TMEM230* is a recently identified gene, and mutations in this gene cause PD in an autosomal dominant manner (Deng et al. 2016). TMEM230 is a protein containing two transmembrane domains. A previous study showed that TMEM230 is present as both a long isoform (isoform 1) and a short isoform (isoform 2), and the latter is abundant in the cell. Two missense mutations, Y92C (Y29C in isoform 2) and R141L (R78L in isoform 2), and two of additional mutations, which add an extra six (W5E) and seven (PG5E) amino acids to the C-terminus, were identified as pathogenic PD-related mutants (Deng et al. 2016). However, referring to *TMEM230* as a PD-causing gene should be approached cautiously, because most sequencing studies with PD cohorts failed to find additional cases with these mutations, with one exception that identified a new pathogenic mutation of *TMEM230* (Baumann et al. 2017). A TMEM230 functional study suggested that the protein regulates Rab8-mediated secretory vesicle trafficking and retromer trafficking (Kim et al. 2017). In addition, a recent study reported that TMEM230 is a novel regulator of angiogenesis in zebrafish (Carra et al. 2018).

The most common cause of familial PD is mutation in leucine-rich repeat kinase 2 (LRRK2), and the LRRK2 protein contains functional GTPase and kinase domains and regulates autophagy, neurite outgrowth and vesicle trafficking (Rideout 2017; Seol 2010). LRRK2 has been reported to interact with other PD-causing proteins such as α-synuclein (Guerreiro et al. 2013) and parkin (Smith et al. 2005). Especially, expression of WT or pathogenic G2019S LRRK2 has been reported to accelerate neuropathological phenotypes developed in pathogenic α-synuclein transgenic mice (Lin et al. 2009). In contrast, another study reported that G2019S, but not WT LRRK2, promotes formation of α-synuclein inclusions (Volpicelli-Daley et al. 2016). These studies suggested that at least pathogenic G2019S LRRK2 facilitates pathogenicity of α-synuclein protein aggregation.

In this study, we attempted to elucidate the function of both WT and PD-related mutant TMEM230 proteins in terms of PD pathogenesis and explore the relationship between TMEM230 and LRRK2.

## Materials and methods

### Construction of plasmids containing WT and mutant TMEM23

The gene encoding human TMEM230 isoform 2 was synthesized by RT–PCR with primers TMEM WT-F, CCGGATCCGAATTCATGATGCCGTCCCGTACCAAC and TMEM WT-R, GGCTCGAGCTAGTCATCAAAGTCTGGAATG, using clone BKU008111 from the Korea Human Gene Bank (Daejeon, Korea) as a template. The amplification product was digested with *EcoRI* and *XhoI* and cloned into pcDNA3.1 with a Flag tag. The R78L (isoform 2 of R141L) mutation was introduced by *in vitro* site-directed mutagenesis using primers, TMEM R78L-F, CAAAGGGGGGGCAGACCtGGCCGTTCCAG and TMEM R78L-R, CTGGAACGGCCaGGTCTGCCCCCCCTTTG (The lowercase letter is the mutated site.). The PG5E (isoform 2 of *120PGext*5, (Deng et al., 2016)) mutation was introduced by replacing a WT DNA fragment with the corresponding mutant DNA fragment synthesized by PCR with primers, the TMEM230 WT-F and TMEM230 PG5E-R, GGCTCGAGTCAGCTATGGGGTGGGTGCCCGGGGTCATCAAAGTCTGG. To construct GFP fusion of these TMEM230 clones, each PCR product was digested with *BamHI* and *XhoI* and cloned into an empty GFP fusion vector. The DNA sequences of all clones were confirmed by sequencing, and expression of the expected protein was confirmed by western blotting.

The following antibodies were used: anti-LRRK2 (MJFF2 Abcam, Cambridge, MA, USA; ab133474, 1:1000), anti-Flag Tag (Cell Signal Technology, Danvers, MA, USA; 8146 or 2368K, 1:1000), anti-Myc (9E11; Santa Cruz Biotechnology, Dallas, TX, USA: sc-47694, 1:500), anti-GFP (B-2; Santa Cruz, Dallas, TX, USA: sc-9996), anti-β-actin (Santa Cruz, Dallas, TX, USA; sc-47778, 1:1000), anti-Alix (Santa Cruz, Dallas, TX, USA; sc-53540, 1:1000), anti-TSG101 (4A10 Abcam, Cambridge, MA, USA; ab83, 1:1000), anti-Rab1A (C19 Santa Cruz, Dallas, TX, USA; sc-311, 1:1000), anti-Rab5 (D-11 Santa Cruz, Dallas, TX, USA; sc-46692, 1:1000), anti-Rab7 (D95F2 Cell Signal Technology, Danvers, MA, USA; 9367, 1:1000), anti-Rab11 (D4F5 Cell Signal Technology, Danvers, MA, USA; 5589, 1:1000) and anti-Rab8A (63-BJ Santa Cruz, Dallas, TX, USA; sc-81909, 1:500).

### Cell culture, transfection and fluorescence microscopy

We used HEK 293T and human and murine dopaminergic neuronal cell lines, SH-SY5Y and SN4741, respectively. 293T and SH-SY5Y cells were maintained in DMEM containing 10% fetal bovine serum at 37°C and 5% CO_2_. SN4741 cells were obtained from Dr. HJ Son (Ehwa Women’s University) and maintained in the same medium at 33°C.

For plasmid transfection, Lipofectamine 2000 (Invitrogen, Carlsbad, CA, USA; 11668-019), Lipofectamine LTX (Invitrogen, 15338-100) and LipoD293™ (SignaGen, Rockville, *MD, USA; SL100668)* were used for HEK293T, SH-SY5Y, and SN4741 cells, respectively, as recommended by the manufacturer. When needed, cycloheximide (20 μg/ml) was added to the cells 40 h after transfection.

For microscopy, SN4741 cells were transfected with GFP-tagged TMEM230 WT or mutant plasmids and observed under a FLoid™ Cell Imaging Station (Thermo Fisher Scientific, Waltham, MA, USA). A representative image was selected from each set.

### GFP trap assay

HEK293T cells (5 × 10^5^ cells) were seeded in a 60 mm dish and transiently co-transfected with LRRK2 WT (9 μg) and GFP, GFP-TMEM230 WT, -TMEM230 R78L, or -TMEM230 PG5E (1 μg) using Lipofectamine 2000. Then, a GFP-trap assay was performed using the GFP-Trap®_A beads (Chromotek, Planegg-Martinsried, Germany; gta-20) according to the manufacturer’s instructions. Samples were subsequently analyzed by SDS-PAGE and western blotting using the indicated antibodies.

### Western blotting

Cells were harvested and lysed with 1 × sample loading buffer. Total cell lysates, without any centrifugation step, were loaded on to a 12% SDS-PAGE gel or a 4–15% gradient gel (Bio-Rad, Hercules, CA, USA; 456-1086), transferred to a nitrocellulose membrane, and analyzed with the indicated antibodies.

### *In vitro* kinase assay

A recombinant GST-TMEM230 WT protein purified from *E. coli* was used as the substrate in an *in vitro* kinase assay with a ΔN-GST-LRRK2 G2019S recombinant protein (Invitrogen, PV4881) as previously described (Ho et al. 2015).

### FACS analysis

SN4741 cells (1.3 × 10^5^ cells) were seeded in 60 mm dishes and transfected with GFP, GFP-TMEM230 WT, -TMEM230 R78L, or -TMEM230 PG5E (8 μg). When indicated, MPP^+^ iodide (D048, 1 mM, Sigma-Aldrich, Saint Louis, MO, USA) was added to the cells at 24 h after transfection, and incubated further for 24 h. Then, the cells were washed with PBS, detached with TrypLE™ Express (Gibco, Gaithersburg, MD, USA; 12604-013), and washed with FACS buffer (10% FBS in PBS). Finally, the cells were suspended in 400 μL of fresh FACS buffer containing propidium iodide (PI, 10 μg/ml, Dojindo Molecular Technologies, Inc., Rockville, *MD, USA*; P346) to detect dead cells and analyzed with a FACS Aria™III (BD Biosciences, Denmark).

### Statistical analysis

The densities of the bands in the western blots were analyzed using *Multi*-*Gauge* v 3.0 software (Fuji photo Film, Tokyo, Japan). Data were analyzed and diagramed with Prism 6.0 (GraphPad, La Jolla, CA, USA). Data are the mean ± standard error of the mean (SEM). The statistical tests used for data analysis are described in each figure legend.

## Results and discussion

### Overexpression of TMEM230 generates aggregates

**Figure 1.**
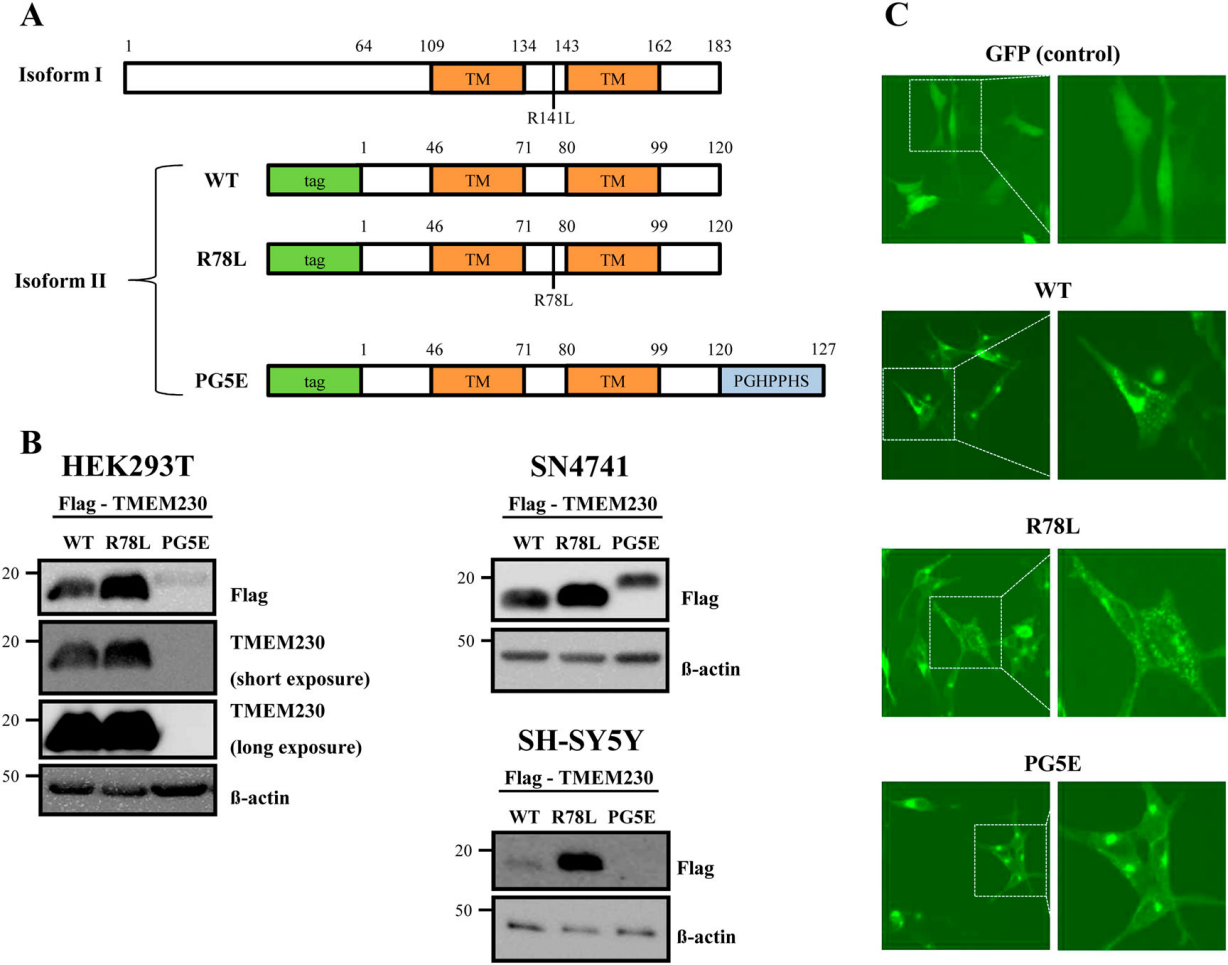
Expression of TMEM230. (A) A scheme of TMEM230 WT and the tested mutants in isoforms 1 and 2. The locations of two putative transmembrane domains (TM), in R78L and PG5E (PGHPPHS) are indicated. (B) Western blot images of Flag-TMEM230 WT, -TMEM230 R78L or -TMEM230 PG5E expressed in SN4741, HEK293T and SH-SY5Y cells. (C) Fluorescence microscopic images of SN4741 cells expressing GFP, GFP-TMEM230 WT, -TMEM230 R78L or -TMEM230 PG5E.

To identify the function of WT TMEM230 and compare it to the two mutant TMEM230 proteins, R78L and PG5E, we constructed plasmids to overexpress these proteins with Flag or GFP tag and transfected them into SN4741 murine dopaminergic cells (Son et al. 1999). We tested TMEM230 isoform 2 instead of isoform 1 because isoform 2 is more abundant in cells (Deng et al. 2016). Expression of the TMEM proteins was confirmed by western blotting and fluorescence analysis (Figure 1(B and C)). Unlike a previous study, which showed no distinct difference in protein levels between WT and the mutants (Deng et al. 2016), the R78L and PG5E proteins consistently showed higher and lower expression, respectively, than the WT proteins in all three tested cell types (HEK293T, SH-SY5Y and SN4741) (Figure 1(B)). Interestingly, PG5E was detected only by the anti-Flag antibody, but not by the anti-TMEM230 antibody (Figure 1(B)). This might be due to either its relatively low expression or a conformation difference of PG5E (Figure 1(B)).

Fluorescence images of transfected cells showed that expression of GFP-WT and -mutant proteins resulted in formation of puncta or aggregates whereas expression of the control GFP showed even distribution (Figure 1(C)), as previously described (Deng et al. 2016). Overall, there was no distinct difference between WT and mutants although cells expressing puncta were predominantly observed in PG5E. The formation of puncta might be due to an intrinsic property of TMEM230 proteins rather than their overexpression because both overexpressed and endogenous TMEM230 WT have exhibited similar pattern of puncta (Deng et al. 2016).

We also tested whether low protein level of PG5E (Figure 1) is due to formation of its SDS-resistant aggregates which might be present at the top of the protein gel, although the possibility is low since the cells were lyzed with the sample loading buffer containing 2% SDS. The western blot result shows no detection at the top of the gel in both WT and the mutant lanes, indicating absence of SDS-resistant aggregates (data not shown).

### The R78L and PG5E mutations impair TMEM230 protein stability

**Figure 2.**
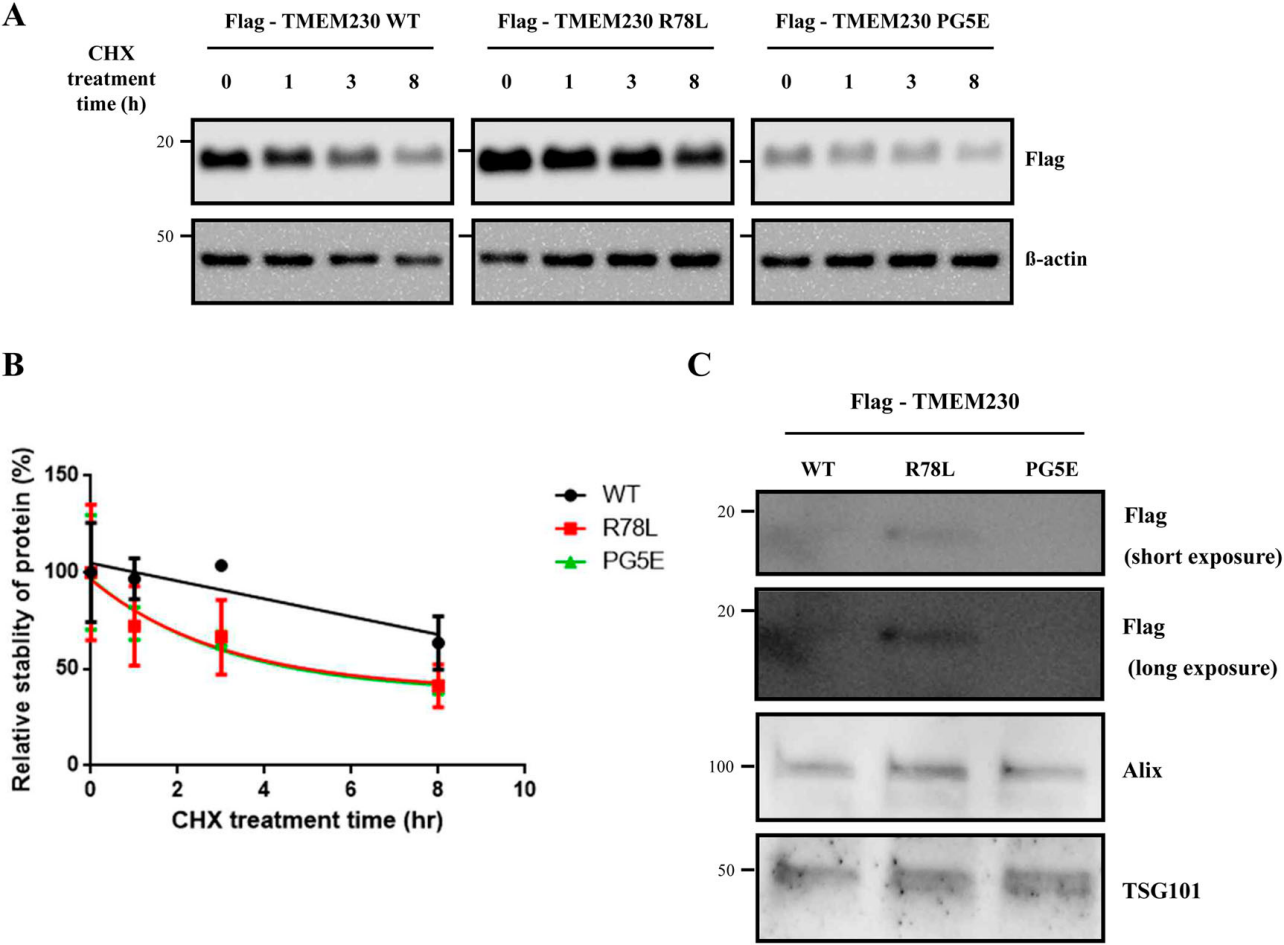
Stability of Flag-TMEM230 WT, -TMEM230 R78L or -TMEM230 PG5E proteins. (A) SN4741 cells were transfected with the indicated TMEM230 plasmids for 40 h and then treated with cycloheximide (CHX) for 0, 1, 3 and 8 h. Each TMEM230 protein level is analyzed by western blot. *n* = 3. (B) Relative level of TMEM230 proteins after cycloheximide treatment. (C) Western blot analysis of TMEM230 protein secreted in culture media. Alix and TSG101 were used as loading controls of media. A representative image is shown from three separate experiments (A, C).

We observed different protein levels in cells transfected with WT or mutant *TMEM230* genes although they were cloned into the same site of the same vector. Thus, we wondered whether the difference in protein levels might be due to variations in protein stability. Therefore, we treated SN4741 cells expressing each TMEM230 protein with cycloheximide for the indicated times to inhibit protein synthesis, and TMEM230 protein was detected by western blotting (Figure 2(A)). The results demonstrated that the stability of both mutants, R78L and PG5E, is impaired when compared to WT, and the mutants show reduced, albeit similar to each other, stability (Figure 2(A and B)). This result is inconsistent with the lower expression of PG5E compared to that of R78L (Figures 1(B) and 2(A), time 0). We reasoned that this might be due to higher secretion of PG5E compared to that of R78L; therefore, we evaluated their secretion levels in culture medium. As shown in Figure 2(C), the secreted levels of these proteins were as follows: R78L > WT > PG5E, which is the same order for the intracellular amounts. Therefore, the difference in cellular protein levels between R78L and PG5E was not due to a difference in secretion.

PG5E contains replacement of TAG by CCCGGG compared to WT. The lower expression of PG5E could be due to such a base difference of PG5E, which may function as a transcription/translation inhibitory sequence to change expression level of PG5E protein. Otherwise, the observed differences in their expression levels might be due to a cloning artifact because a previous report showed similar protein levels of WT and PG5E TMEM230 in HEK293T cells (Deng et al. 2016). If the cellular levels of the WT and mutants proteins are similar as previously described, the lower stabilities of the pathogenic mutants, when compared to WT, might be involved in the mechanism of PD pathogenesis caused by TMEM230.

### Neurotoxicity of TMEM230

**Figure 3.**
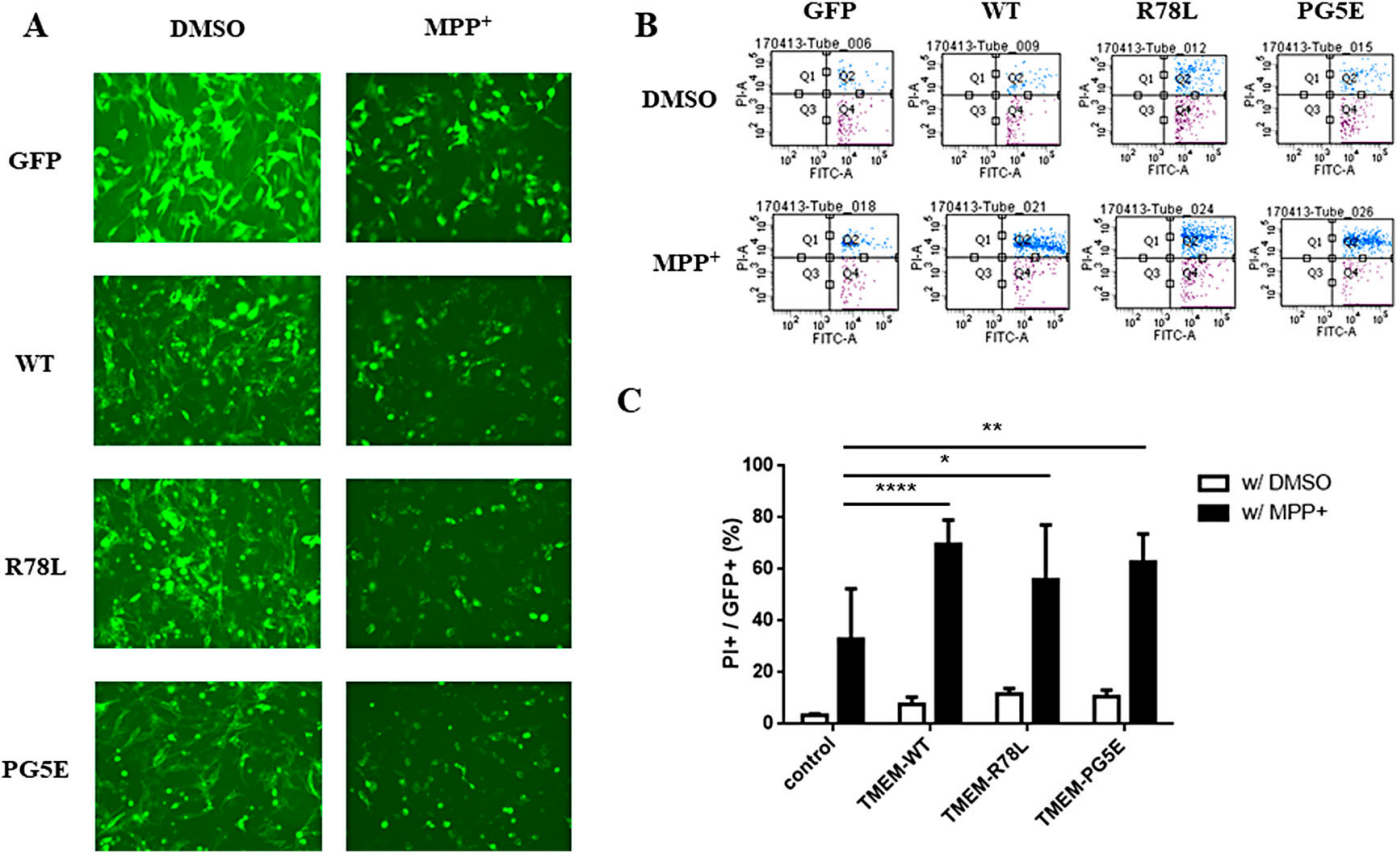
FACS analysis of SN4741 cells expressing GFP, GFP-TMEM230 WT or GFP-TMEM230 mutant proteins after treatment with or without MPP^+^. (A) Fluorescence images of cells expressing GFP proteins after MPP^+^ treatment. (B) Scatter plots showing the distribution of GFP and PI staining for each group. (C) Quantitative analysis of the percentage of dead cells by FACS analysis. The data were analyzed by *two*-*way ANOVA* followed by *Tukey’s post-hoc* test (*n* = 6). There was no significant difference among the cells transfected with the empty vector and TMEM genes and treated with DMSO. *p < 0.05, **p < 0.01, ****p < 0.0001.

Expression of pathogenic mutants of dominantly inherited PD-related proteins, such as α–synuclein and LRRK2, tends to generate aggregates and promote neurotoxicity (Smith et al. 2005; Burre et al. 2015). Figures 1(C) and 3(A) show that the overexpression of TMEM230 leads to aggregates. To test if the WT or pathogenic mutant TMEM230 proteins are neurotoxic on the presence of PD-causing chemicals, SN4741 cells expressing WT or pathogenic mutant TMEM230 proteins were treated with MPP^+^, a well known PD-causing chemical, and dead cells were counted by FACS after PI staining. Figure 3 shows that, without MPP^+^ treatment, overexpression of WT and mutant TMEM230 is weakly neurotoxic when compared to the vector control; however, the differences were not significant. Upon MPP^+^ treatment, both the WT and mutant proteins caused significantly more cell death than the vector control; however, there was no significant difference between cells expressing the WT and mutant proteins (Figure 3(C)). As expected, MPP^+^ treatment itself increased cell death (Figure 3(C)).

It has been reported that the expression of proteins encoded by PD-causing genes is neurotoxic and that toxicity increase in the presence of oxidative stress or PD-causing chemicals (Heo et al. 2010). In addition, the expression of pathogenic mutants of dominantly inherited PD-causing genes, such as LRRK2 or α-synuclein, is more toxic than the expression of the corresponding WT proteins (Ostrerova-Golts et al. 2000; Smith et al. 2005; Heo et al. 2010; Xiao et al. 2015). Although TMEM230 has been also reported as a dominantly inherited PD-causing gene, there was no difference in the neurotoxicity of WT and the pathogenic mutant proteins with and without insult-causing chemical treatment. However, the neurotoxicity of both the WT and mutant proteins increased in the presence of MPP^+^ compared to that of the vector control. These results suggested that TMEM230 itself, but not the pathogenic mutations, is related to PD pathogenesis.

Since the first report of *TMEM230* as a PD-causing gene (Deng et al. 2016), several studies have been attempted to confirm the pathogenic mutations in various ethnic cohorts of PD patients. However, most studies failed to identify any reported or unreported PD-specific mutation in TMEM230 (Fan et al. 2017; Giri et al. 2017; Ibanez et al. 2017; Wei et al. 2018) with one exception (Baumann et al. 2017). Thus, there is concern whether TMEM230 is a real PD-causing gene (Mandemakers et al. 2017). Our results showed that TMEM230 is neurotoxic upon MPP^+^ treatment, although there is no difference between the WT and mutants. This puzzling issue might be elucidated in a future study in which cells expressing TMEM230 are treated with PD-causing chemicals other than MPP^+^.

### Interaction between TMEM230 and LRRK2

Among the various functions of LRRK2, encoded by another prevalent PD-causing gene, is vesicle trafficking (Shin et al. 2008; Piccoli et al. 2014). TMEM230 has been reported to function in Rab8a-mediated secretory vesicle trafficking and retromer trafficking (Kim et al. 2017). Therefore, we tested whether LRRK2 mediates or regulates TMEM230 or *vice versa*.

**Figure 4.**
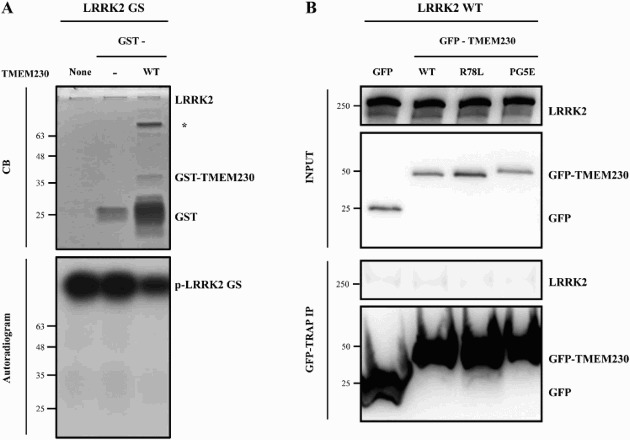
TMEM230 neither interacts with LRRK2 nor is a kinase substrate for LRRK2. A. LRRK2 kinase assay. Purified GST or GST-TMEM230 WT proteins were used as the substrate and incubated with [γ-^32^P]-ATP and recombinant GST-ΔN LRRK2 G2019S (GS) proteins in a LRRK2 kinase assay. CB: Coommasie blue; *: non-specific band. B. A GFP- trap assay for GFP-TMEM230 and LRRK2. HEK293T cells co-transfected with LRRK2 WT and GFP, GFP-TMEM230 WT, -TMEM230 R78L or -TMEM230 PG5E were used in the GFP-Trap assay. Immunoprecipitates were analyzed with anti-LRRK2 and anti-GFP antibodies.

First, we investigated whether TMEM230 is a substrate for LRRK2 kinase activity. In an *in vitro* LRRK2 kinase assay, purified recombinant GST-TMEM230 WT protein was not phosphorylated even after overexposure, although LRRK2 itself was strongly autophosphorylated (Figure 4(A)). Then we tested for an interaction between LRRK2 and TMEM230 (WT and pathogenic mutants) using a GFP-trap assay (Lee et al. 2017). However, no interaction between LRRK2 and GFP-TMEM230 proteins was detected (Figure 4(B)). Co-localization analysis of LRRK2 and TMEM230 by immunofluorescence microscopy also showed no signal overlap (data not shown).

These results suggest that LRRK2 and TMEM230 do not interact with each other and independently regulate vesicle trafficking.

### Expression of TMEM230 does not regulate Rab proteins

**Figure 5.**
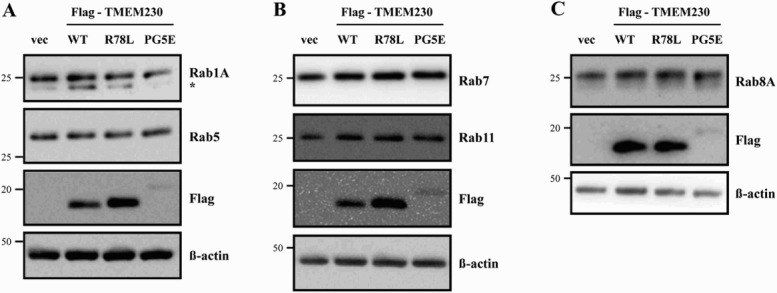
Effect of TMEM230 expression on Rab protein levels. Total lysates of SN4741 cells expressing Flag-TMEM230 WT, -TMEM230 R78L or -TMEM230 PG5E were prepared, loaded into three separate gels (A, B and C) and analyzed by the indicated antibodies by western blotting (*n* = 3). vec: control vector. *: non-specific band.

It was previously reported that TMEM230 regulates Rab8a-mediated secretory vesicle trafficking and retromer trafficking (Kim et al. 2017). Therefore, we investigated whether TMEM230 WT and mutant proteins effect on the levels of various Rab proteins. We tested several endosome markers, including Rab5, 7 and 11. There was no significant difference among cells transfected with the empty vector and TMEM genes after normalization to β- actin (Figure 5). We also tested the levels of other Rab proteins, such as Rab 1A (an ER-Golgi traffic marker) and 8A (a secretory vesicle trafficking marker). Again, there was no clear difference in Rab1A and 8A protein levels between cells transfected with vector and those expressing the TEMEM230 WT and mutant proteins.

Taken together, this study showed that TMEM230 protein is neurotoxic in cells treated with MPP^+^, although there is no difference in the neurotoxicity of WT and mutants and the pathogenic mutant proteins are relatively unstable compared to WT. To determine whether TMEM230 is a real PD-causing gene, further studies are necessary.

## References

[CIT0001] Baumann H, Wolff S, Munchau A, Hagenah JM, Lohmann K, Klein C. 2017 Evaluating the role of TMEM230 variants in Parkinson’s disease. Parkinsonism Relat Disord. 35:100–101. doi: 10.1016/j.parkreldis.2016.12.015 28017548

[CIT0002] Burre J, Sharma M, Sudhof TC. 2015 Definition of a molecular pathway mediating alpha-synuclein neurotoxicity. J Neurosci. 35:5221–5232. doi: 10.1523/JNEUROSCI.4650-14.2015 25834048 PMC4380997

[CIT0003] Carra S, Sangiorgio L, Pelucchi P, Cermenati S, Mezzelani A, Martino V, Palizban M, Albertini A, Gotte M, Kehler J, et al. 2018 Zebrafish Tmem230a cooperates with the Delta/Notch signaling pathway to modulate endothelial cell number in angiogenic vessels. J Cell Physiol. 233:1455–1467. doi: 10.1002/jcp.26032 28542953

[CIT0004] Deng HX, Shi Y, Yang Y, Ahmeti KB, Miller N, Huang C, Cheng L, Zhai H, Deng S, Nuytemans K, et al. 2016 Identification of TMEM230 mutations in familial Parkinson’s disease. Nat Genet. 48:733–739. doi: 10.1038/ng.3589 27270108 PMC6047531

[CIT0005] Fan TS, Lin CH, Lin HI, Chen ML, Wu RM. 2017 Lack of TMEM230 mutations in patients with familial and sporadic Parkinson’s disease in a Taiwanese population. Am J Med Genet B Neuropsychiatr Genet. 174:751–756. doi: 10.1002/ajmg.b.32576 28766910

[CIT0006] Giri A, Mok KY, Jansen I, Sharma M, Tesson C, Mangone G, Lesage S, Bras JM, Shulman JM, Sheerin UM, et al. 2017 Lack of evidence for a role of genetic variation in TMEM230 in the risk for Parkinson’s disease in the caucasian population. Neurobiol Aging. 50:167.e11–167.e13. doi: 10.1016/j.neurobiolaging.2016.10.004 PMC5812479 27818000

[CIT0007] Guerreiro PS, Huang Y, Gysbers A, Cheng D, Gai WP, Outeiro TF, Halliday GM. 2013 LRRK2 interactions with alpha-synuclein in Parkinson’s disease brains and in cell models. J Mol Med (Berl). 91:513–522. doi: 10.1007/s00109-012-0984-y 23183827 PMC3611031

[CIT0008] Heo HY, Park JM, Kim CH, Han BS, Kim KS, Seol W. 2010 LRRK2 enhances oxidative stress-induced neurotoxicity via its kinase activity. Exp Cell Res. 316:649–656. doi: 10.1016/j.yexcr.2009.09.014 19769964

[CIT0009] Ho DH, Kim H, Kim J, Sim H, Ahn H, Kim J, Seo H, Chung KC, Park BJ, Son I, et al. 2015 Leucine-Rich Repeat Kinase 2 (LRRK2) phosphorylates p53 and induces p21(WAF1/CIP1) expression. Mol Brain. 8:54. doi: 10.1186/s13041-015-0145-7 26384650 PMC4575451

[CIT0010] Ibanez L, Dube U, Budde J, Black K, Medvedeva A, Davis AA, Perlmutter JS, Benitez BA, Cruchaga C. 2017 TMEM230 in Parkinson’s disease. Neurobiol Aging. 56:212.e1–212.e3. doi: 10.1016/j.neurobiolaging.2017.03.014 PMC5526081 28457580

[CIT0011] Kim MJ, Deng HX, Wong YC, Siddique T, Krainc D. 2017 The Parkinson’s disease-linked protein TMEM230 is required for Rab8a-mediated secretory vesicle trafficking and retromer trafficking. Hum Mol Genet. 26:729–741. 28115417 10.1093/hmg/ddw413 PMC6251589

[CIT0012] Lee Y, Kim SG, Lee B, Zhang Y, Kim Y, Kim S, Kim E, Kang H, Han K. 2017 Striatal transcriptome and interactome analysis of Shank3-overexpressing mice reveals the connectivity between Shank3 and mTORC1 signaling. Front Mol Neurosci. 10:201. doi: 10.3389/fnmol.2017.00201 28701918 PMC5487420

[CIT0013] Lin X, Parisiadou L, Gu XL, Wang L, Shim H, Sun L, Xie C, Long CX, Yang WJ, Ding J, et al. 2009 Leucine-rich repeat kinase 2 regulates the progression of neuropathology induced by Parkinson’s-disease-related mutant alpha-synuclein. Neuron. 64:807–827. doi: 10.1016/j.neuron.2009.11.006 20064389 PMC2807409

[CIT0014] Mandemakers W, Quadri M, Stamelou M, Bonifati V. 2017 TMEM230: how does it fit in the etiology and pathogenesis of Parkinson’s disease? Mov Disord. 32:1159–1162. doi: 10.1002/mds.27061 28568905

[CIT0015] Martin I, Dawson VL, Dawson TM. 2011 Recent advances in the genetics of Parkinson’s disease. Annu Rev Genomics Hum Genet. 12:301–325. doi: 10.1146/annurev-genom-082410-101440 21639795 PMC4120236

[CIT0016] Ostrerova-Golts N, Petrucelli L, Hardy J, Lee JM, Farer M, Wolozin B. 2000 The A53T alpha-synuclein mutation increases iron-dependent aggregation and toxicity. J Neurosci. 20:6048–6054. 10934254 10.1523/JNEUROSCI.20-16-06048.2000 PMC6772599

[CIT0017] Piccoli G, Onofri F, Cirnaru MD, Kaiser CJ, Jagtap P, Kastenmuller A, Pischedda F, Marte A, von Zweydorf F, Vogt A, et al. 2014 Leucine-rich repeat kinase 2 binds to neuronal vesicles through protein interactions mediated by Its C-terminal WD40 Domain. Mol Cell Biol. 34:2147–2161. doi: 10.1128/MCB.00914-13 24687852 PMC4054300

[CIT0018] Rideout HJ, editor 2017 Leucine Rich Repeat Kinase 2 (LRRK2). Vol. 14, Advances in neurobiology. Springer. doi: 10.1007/978-3-319-49969-7

[CIT0019] Seol W. 2010 Biochemical and molecular features of LRRK2 and its pathophysiological roles in Parkinson’s disease. BMB Rep. 43:233–244. doi: 10.5483/BMBRep.2010.43.4.233 20423607

[CIT0020] Shin N, Jeong H, Kwon J, Heo HY, Kwon JJ, Yun HJ, Kim CH, Han BS, Tong Y, Shen J, et al. 2008 LRRK2 regulates synaptic vesicle endocytosis. Exp Cell Res. 314:2055–2065. doi: 10.1016/j.yexcr.2008.02.015 18445495

[CIT0021] Smith WW, Pei Z, Jiang H, Moore DJ, Liang Y, West AB, Dawson VL, Dawson TM, Ross CA. 2005 Leucine-rich repeat kinase 2 (LRRK2) interacts with parkin, and mutant LRRK2 induces neuronal degeneration. Proc Natl Acad Sci USA. 102:18676–18681. doi: 10.1073/pnas.0508052102 16352719 PMC1317945

[CIT0022] Son JH, Chun HS, Joh TH, Cho S, Conti B, Lee JW. 1999 Neuroprotection and neuronal differentiation studies using substantia nigra dopaminergic cells derived from transgenic mouse embryos. J Neurosci. 19:10–20. 9870933 10.1523/JNEUROSCI.19-01-00010.1999 PMC6782395

[CIT0023] Volpicelli-Daley LA, Abdelmotilib H, Liu Z, Stoyka L, Daher JP, Milnerwood AJ, Unni VK, Hirst WD, Yue Z, Zhao HT, et al. 2016 G2019S-LRRK2 expression augments alpha-synuclein sequestration into inclusions in neurons. J Neurosci. 36:7415–7427. doi: 10.1523/JNEUROSCI.3642-15.2016 27413152 PMC4945663

[CIT0024] Wei Q, Ou R, Zhou Q, Chen Y, Cao B, Gu X, Zhao B, Wu Y, Song W, Shang HF. 2018 TMEM230 mutations are rare in Han Chinese patients with autosomal dominant Parkinson's disease. Mol Neurobiol. 55:2851–2855. doi: 10.1007/s12035-017-0542-2 28455698

[CIT0025] Xiao Q, Yang S, Le W. 2015 G2019s LRRK2 and aging confer susceptibility to proteasome inhibitor-induced neurotoxicity in nigrostriatal dopaminergic system. J Neural Transm (Vienna). 122:1645–1657. doi: 10.1007/s00702-015-1438-9 26253900

